# Implementing Diabetes Distress Screening in a Pediatric Endocrinology Clinic Using a Digital Health Platform: Quantitative Secondary Data Analysis

**DOI:** 10.2196/65107

**Published:** 2025-02-06

**Authors:** Nicole A Kahhan, Larry A Fox, Matthew Benson, Susana R Patton

**Affiliations:** 1 Nemours Children's Health- Jacksonville, 807 Children's Way, Jacksonville, FL, 32207, United States, 1 904-697-3600

**Keywords:** type 1 diabetes, diabetes mellitus, type 1, pediatric, child, children, youth, parents, diabetes distress, eHealth, screening, digital health, diabetes, diabetic, type 1, DM, T1D, endocrinology, alert, best practice alert, BPA, patient education

## Abstract

**Background:**

Type 1 diabetes (T1D) management requires following a complex and constant regimen relying on child or caregiver behaviors, skills, and knowledge. Psychological factors such as diabetes distress (DD), depression, and burnout are pertinent considerations in the treatment of pediatric T1D. Approximately 40% of youth and 61% of caregivers experience DD. Implementation of DD screening as part of clinical best practice is recommended and may facilitate treatment referral, perhaps leading to improved health or well-being for youth with T1D and their caregivers. By building on existing institutional infrastructure when available, screening via digital health platforms (applications, or “apps”) may allow for timely screening of, and response to, DD.

**Objective:**

This work details the creation, implementation, and refinement of a process to screen for DD in youth and their caregivers in the context of routine T1D care using a digital health platform.

**Methods:**

DD screening was implemented in an outpatient endocrinology clinic over 1 year as part of a larger screen-to-treat trial for children aged 8‐12.99 years and their caregivers. Validated measures were sent via digital health platform to be completed prior to the clinic visit. Results were initially reviewed manually, but a digital best practice alert (BPA) was later built to notify staff of elevated scores. Families experiencing DD received resources sent via the digital health platform. For this secondary analysis, child demographics and glycated hemoglobin A_1c_ (HbA_1c_) were collected.

**Results:**

During the screening period, absolute completion rates were 36.78% and 38.83%, with adjusted screening rates at 52.02% and 54.48%, for children and caregivers, respectively. A total of 21 children (mean HbA_1c_ 8.04%, SD 1.39%) and 26 caregivers (child mean HbA_1c_ 8.04%, SD 1.72%) reported elevated DD. Prior to BPA development, resources were sent to all but 1 family. After BPA implementation, all families were sent resources.

**Conclusions:**

Early findings indicate that DD education, screening, and response can be integrated via digital platforms in a freestanding outpatient endocrinology clinic, thereby facilitating timely treatment referral and provision of resources for those identified with distress. Notably, in the observed 1-year screening period, screening rates were low, and barriers to implementation were identified. While some implementation challenges were iteratively addressed, there is a need for future quality improvement initiatives to improve screening rates and the identification of, or response to, DD in our pediatric patients and their families.

## Introduction

Rates of type 1 diabetes (T1D) in youth aged 19 years or younger have been increasing in recent years, from approximately 1.48 per 1000 youth in 2001 to 2.15 per 1000 youth in 2017 [1]. Rates increased at the highest levels in non-Hispanic White and non-Hispanic Black children [1]. Health-promoting management of T1D requires following a complex and constant treatment regimen with tasks relying on child and caregiver behaviors, skills, and knowledge [2]. Given the complexity and constancy of diabetes management, it is not surprising that psychological factors such as distress, depression, anxiety, and burnout are highlighted as pertinent to consider in the management of pediatric T1D [2-4].

Diabetes distress (DD) is the “emotional distress that results from living with diabetes and the burden of relentless daily self-management” that can be seen across the life span, as well as in caregivers of those with diabetes [5]. It occurs at rates of approximately 25% in adults with T1D [6]. In children aged 8‐12 years, as many as 40% of youth and 61% of their parents or caregivers experience at least some DD [7]. Notably, DD is occurring at higher rates, on average, than depression in pediatric populations with diabetes [8,9]. Increased levels of DD relate to deficits in diabetes self-management behaviors, increased glycated hemoglobin A_1c_ (HbA_1c_), and negative impacts on mental health and well-being [4,6]. DD differs from burnout, defined as the physical or emotional exhaustion associated with continuous DD and management needs, and depression, although these can co-occur [10,11]. Implementation of DD screening (and subsequently, treatment of DD) as part of clinical best practice may facilitate treatment referral and could lead to improved health and well-being for youth and their caregivers [6].

The American Diabetes Association (ADA) Standards of Care in Diabetes recommends DD screening starting at 8 years of age, with the parent, child, and adolescent versions of the Problem Areas in Diabetes (PAID) highlighted as validated assessment tools in this domain [2]. Similarly, the International Society for Pediatric and Adolescent Diabetes (ISPAD) recommends that age-appropriate and validated assessment tools be used routinely to monitor and guide conversations specific to the psychosocial well-being of all youth with diabetes as well as their caregivers [4]. Despite these recommendations, 1 recent publication reported that less than half of surveyed pediatric diabetes clinics screened for mental health problems of any kind using a validated tool [12]. In contrast, another survey of T1D exchange participants reported that 96% of pediatric centers included use at least 1 standardized measure of patient reported symptoms or needs. However, measures included in this study were more broadly inclusive of mental health, transition readiness, and structural determinants of health, among other domains, with <30% of centers reporting screening for DD [13].

In line with best practice recommendations, we sought to implement standardized DD screening for youth aged 8‐12 years and their caregivers in an outpatient endocrinology clinic in a large, freestanding, pediatric medical center. Screening was completed using validated surveys sent prior to children’s clinical visit via digital health platform. Thus, by building on existing infrastructure, it was possible to conduct DD screening and deliver a response to elevated scores using the institutional app, which we anticipated would be a highly scalable process.

## Methods

### Participants

This project occurred in the pediatric endocrinology clinic at Nemours Children’s Health-Jacksonville, which serves more than 1000 children with T1D. The reported results focus on screening procedures initiated and tested from April 1, 2022, through March 31, 2023. Children eligible for screening were aged between 8 and 12.99 years, with any diagnosis of diabetes (broadly identified by visit type, because at the time of implementation, the system could not differentiate between T1D, type 2 diabetes, or another diabetes), and able to read and understand English. Adolescents aged 13 years and older were excluded from DD screening because they were already participating in another screening initiative at our institution (depression screening). Eligible parents or caregivers had a child who met the eligibility criteria, were signed up to use the Nemours app for health care management, and were able to read and understand English. The Nemours app is a stand-alone app created by the larger Nemours Children’s Health system. Families were encouraged to sign up for this app beginning in August 2019 to access child health records, manage appointments, message providers, complete paperwork and payments, receive resources, and participate in telehealth visits. At the start of the screening period (April 2022), approximately 68% of families followed in the endocrinology clinic were signed up for the Nemours app, although this increased to 78% by month 12 (Table 1). Of note, 30.7% of families on average who started previsit questionnaires in the app (the “GetReady” process) did not complete their questionnaires and were able to attend clinic visits despite outstanding paperwork.

**Table 1.** Application use data and completion of pre–check-in paperwork over 1-year implementation period.

	Month of screening implementation											
	1	2	3	4	5	6	7	8	9	10	11	12
Percentage of patients seen with active app accounts (enterprise-wide)	51.5	52.4	53.3	54.2	55.2	56.0	56.8	57.6	58.4	59.1	59.7	60.5
Percentage of patients seen with active app accounts (division/location-specific)	68.0	69.1	70.6	71.5	73.0	73.3	74.4	75.3	75.9	76.6	77.5	78.0
Percentage of appointments where GetReady was started but not completed (division/location-specific)	34.9	29.3	24.7	31.6	26.1	30.6	33.9	30.5	30.3	32.1	33.6	30.9

### Ethical Considerations

Given the use of retrospective chart reviews for data collection, the authors obtained institutional review board approval (2057003) for secondary (exempt) research prior to the collection of data. The institutional review board determined based on the methods, proposed analyses, and the researcher’s ability to work with deidentified data that informed consent or assent was not required for this project. Compensation was not provided as part of this secondary research.

### Procedure

In line with standard of care recommendations from the ADA and ISPAD [2,4], and as part of a larger screen-to-treat trial, the pediatric endocrinology clinic at Nemours Children’s Health-Jacksonville, implemented a screening program to detect symptoms of DD in school-aged children and their parents. The clinic used automated processes, a digital health platform, and validated screening tools to minimize any negative impact on clinic flow and to capitalize on the existing system for paperwork completion via the Nemours app. Per the screening protocol, the automated system assigned the DD screening tools to children, aged at least 8 years and younger than 13 years, with a visit type “Diabetes NP (new patient) w/Care Team” or “Diabetes FP (follow up patient) w/Care Team” and the clinic location in Jacksonville, Florida. Children and parents received the DD screeners every 6 months via the Nemours app “GetReady” feature along with other clinic surveys (eg, intake form) up to 10 days before their scheduled clinic visit. Regular reminders to complete paperwork were provided prior to the visit through an automated messaging system. Once completed, the Nemours app automatically scored the DD screeners and uploaded the screener results into the child’s electronic health record (EHR). Initially, each week, a diabetes psychologist would manually review the completed DD screeners and send messages in the EHR to the visit provider when a child or parent had an elevated DD screen. Furthermore, for each elevated DD screen, the psychologist manually sent families a message via the EHR, which (1) thanked the family for completing screening; (2) defined and normalized DD; and (3) listed local resources including community diabetes groups and camps, web-based resources, relevant web pages, ways to access mental health services (including within their institution from psychology and social work providers), and information about the larger screen-to-treat DD trial so that families could reach out to learn more if interested. This resource list was created collaboratively between endocrinology physicians, psychologists, and a licensed clinical social worker assigned part-time to the endocrinology clinic. Eventually, to automate the process more fully, the clinic technology team built a best practice alert (BPA) into the EHR so that clinical providers associated with an upcoming visit and the diabetes psychologist would receive an automated alert flag for elevated DD scores. This feature made it possible for providers to engage in standard of care practices to address elevated DD screening results with families directly during the clinic visit and to include a resource list in their electronic after-visit summary. The psychologist also continued to review screening BPAs and send families a local resource list via an EHR message.

### Measures

We selected 2 validated DD screening tools, the Problem Areas in Diabetes-Child (PAID-C) and the Parent Problem Areas in Diabetes-Child (P-PAID-C) to screen for child and parent symptoms of DD, respectively. The PAID-C is an 11-item survey of DD symptoms specifically designed and validated for children aged 8‐12 years [7]. The PAID-C yields a total score that ranges from 11 to 66, with higher scores reflecting more distress. The P-PAID-C is a 16-item survey of DD symptoms specifically designed and validated for parents of school-aged children [7]. Like the child form, the P-PAID-C yields a single total score. The P-PAID-C total score can range from 16 to 96, and higher scores reflect more distress.

We collected child demographics (eg, age, biological sex, race, and ethnicity) and examined these within the larger eligible clinic population; the subpopulation who participated in the screening program; and the group who had elevated DD screening results. We also collected child HbA_1c_ levels from the visit associated with DD screening captured between April 1, 2022, and March 31, 2023. For children’s HbA_1c_, the clinic uses instruments certified by the National Glycohemoglobin Standardization Program and traceable to reference methods from the Diabetes Control and Complications Trial.

### Data Analyses

We used approved tools to retrieve all EHR data. We report the percentage of eligible families screened for DD out of all eligible families in the clinic population (absolute percentage screened) and the percentage of eligible families screened for DD out of all eligible families with a completed clinic visit between April 1, 2022, and March 31, 2023 (adjusted percentage screened). To analyze these data, we examined the screening rate by each month and the average across the year. We also examined the rate of EHR documentation of follow-up resources being sent to families with elevated screening results. To identify elevated DD, the clinic applied a clinical cut point of ≥41 for children and a cut point of ≥64 for parents or caregivers [7]. Descriptive statistics and HbA_1c_ were examined for both the population who completed DD screening and the families who had elevated screening results. Given that the screening period took place over a 12-month period, some families received and completed the screening measures on more than 1 occasion. If a child or a caregiver was identified as having elevated DD on multiple screenings, he or she was sent resources each time; however, for the purposes of data analysis, only the first elevated screen that also had a clinic visit with an associated HbA_1c_ was included for analyses.

## Results

### Participants

Children who completed any DD screening (eg, child and parent or caregiver completed, child-only completed, and parent or caregiver-only completed) were 55.2% female, 44.8% male, and had a mean age of 10.22 (SD 1.36) years. With respect to their self-reported race, 1.8% were Asian American and Pacific Islander, 0.75% were American Indian or Alaskan Native, 0.8% were Asian Indian, 19.6% were Black or African American, 64.16% were White, 5.0% reported more than 1 race, 8.0% reported other/unspecified, and 1.0% reported “prefer not to say.” For their self-reported ethnicity, 12.1% identified Hispanic/Latinx, 86.4% identified not Hispanic/Latinx, and 1.5% reported “prefer not to say” (Table 2).

**Table 2.** Demographic information.

	Participants, n (%)	
**Ethnicity**		
Hispanic/Latinx	49 (12.1)	
Not Hispanic/Latinx	349 (86.4)	
Prefer not to answer	6 (1.5)	
**Race**		
AAPI^a^	7 (1.8)	
American Indian or Alaskan Native	3 (0.8)	
Asian Indian	3 (0.8)	
Black or African American	78 (19.6)	
White	256 (64.2)	
More than 1 race	20 (5.0)	
Other/unspecified	32 (8.0)	
Prefer not to say	4 (1.0)	
**Age (years)**		
8	70 (14.3)	
9	77 (16.4)	
10	102 (21.8)	
11	117 (25.0)	
12	103 (22.0)	
**Sex**		
Female	223 (55.2)	
Male	181 (44.8)	

a AAPI: Asian American and Pacific Islander.

### Primary Outcomes

#### Screening Completion Rates

During the 1-year screening period, the institutional app system automatically assigned a total of 590 PAID-C questionnaires and 649 P-PAID-C questionnaires to children aged 8‐12.99 years and their caregivers, respectively. A higher number of caregiver questionnaires than pediatric questionnaires were assigned, as some pediatric patients had multiple caregivers associated with their account in the institutional app. Of those, 396 PAID-C and 435 P-PAID-C questionnaires assigned were associated with attended clinic visits. Absolute percentage screened (questionnaire completion out of all assigned) were 36.78% (217/590) and 38.83% (252/649), respectively. Screening rates (questionnaire completion) for those who attended their clinic visits (adjusted percentage screened) for children or caregivers were 52.02% (206/396) and 54.48% (237/435), respectively. Completion rates were relatively stable over the 12 months of DD screening.

#### DD Rates and Resource Provision

In total, 10.2% (21/206) PAID-C and 11.0% (26/237) P-PAID-C surveys scored as elevated during the 1-year screening period, with 1 child and 3 caregivers completing the measure with an elevated score at multiple clinic visits. During this period, 11 child and caregiver dyads scored as elevated on both measures of DD, with 4 of these dyads including a child with a diagnosis of type 2 diabetes or prediabetes, and the remaining dyads with a child diagnosed with T1D. All other elevated screens were present in only a caregiver or a child, who was not part of a parent and child dyad. Of those who were identified as having DD, resources were sent in the app to families in response to 91.7% of elevated PAID-C scores and 100% of elevated P-PAID-C scores; only 1 patient who screened as elevated was not flagged by manual processes and did not receive resources. This occurred before the automated BPA system was put in place. After the BPA was established, all families with elevated parent or child DD scores were sent resources electronically.

#### DD, Demographics, and HbA_1c_

Mean HbA_1c_ was calculated for youth with T1D who also attended the clinic visit associated with the date of elevated DD screening (15 PAID-C and 17 P-PAID-C scores were included). Youth with type 2 diabetes or prediabetes were not included in this subsample. For this subsample including all youth with DD, mean HbA_1c_ was 8.04% (SD 1.39%) and mean child HbA_1c_ for those with caregivers screening elevated for DD was 8.04% (SD 1.72%). This subsample of youth was 68% female (17/25), 32% male (8/25), and had a mean age of 10.4 (SD 1.44) years. With respect to their self-reported race, 8.3% were Black or African American (2/24), 70.8% were White (17/24), 8.3% reported more than 1 race (2/24), and 12.5% reported other/unspecified (3/24). For their self-reported ethnicity, 12.5% identified as Hispanic/Latinx (3/24) and 87.5% identified as not Hispanic/Latinx (21/24).

## Discussion

### Principal Findings

This study details the creation, implementation, and refinement of a process to routinely screen for DD in youth with T1D and their caregivers using a digital health platform. Furthermore, we present descriptive information for those who completed screening. During the 1-year screening period, screening rates for DD were relatively stable, and lower than our initial goals. Approximately 10% of youth and 11% of caregivers who completed screening were identified as having elevated DD. Most of these families were appropriately sent resources via EHR when DD was identified, with 1 patient not flagged prior to an automated BPA being placed. Iterative processes allowed for improvements to be made in the way families with DD were screened and identified using our institutional app, and for resources to be appropriately shared in the families through the digital health platform. Additional suggestions for quality improvement (QI) processes to increase DD screening as well as relevant clinical implications can now be trialed based on findings and lessons learned during this initial 1-year screening period.

### Challenges with Screening Implementation

We identified several challenges to screening families using our institutional app, some that were corrected and others that inform future QI initiatives and clinical research. First, while the automated system was coded to assign the DD screening tools to children aged at least 8 years and those younger than 13 years, for a brief period the questionnaires were incorrectly sent to all pediatric patients or parents seen for diabetes associated visit types in endocrinology. Once identified, this error was corrected. However, the error reoccurred following a later system update and because the system assigns questionnaires at the time an appointment is scheduled (sometimes 6 or more months in advance), the clinic experienced a backlog of incorrectly assigned questionnaires intermittently throughout the first 9 months of the screening period. For the current analyses, children and parent or caregivers who incorrectly received the questionnaires due to age were not included. Nevertheless, the error had clinical implications in that some children and parents or caregivers who were outside of the PAID-C normative age range were identified as distressed and sent electronic resources in line with our procedures.

Second, with our institutional app and its supporting automated system, we could only assign the DD screening tools to children aged 8‐12.99 years (and their caregiver) with a visit type “Diabetes NP w/Care Team” or “Diabetes FP w/Care Team”. These visit types are not coded to differentiate between different diabetes diagnoses. Although DD is also observed in persons with type 2 diabetes, the screening tools we used are not validated for families of youth with type 2 diabetes. In the 1-year screening period, there were 4 elevated PAID-C surveys and 4 elevated P-PAID-C surveys associated with youth with type 2 diabetes or prediabetes. These could represent false-positive results. Thus, if using an automated system to assign a clinical screening tool, it may be important to identify a solution for assigning screeners with greater specificity.

Third, while the initial system for manually reviewing screening results and sending electronic resources to families who had an elevated screen was generally effective, 1 child was not immediately identified and therefore did not receive resources in response to his or her elevated score in a timely manner. Although this represented <5% of total population that screened as elevated, it highlighted the need for an automated BPA process in the EHR to promote greater accuracy and improve response time when sending resources to families. Unfortunately, upon implementing the automated BPA, we identified a new problem, as clinical providers had the ability to close the BPAs without sending families electronic resources. Thus, the lesson learned was also the value of providing ongoing provider education about screening processes in clinic so that these alerts could be appropriately responded to.

Fourth, while 73.6% of families in the endocrinology division were signed up for the Nemours app during the screening year, 30.6% of families who started the “GetReady” paperwork did not complete it before their appointment, thus limiting the number of families screened for DD. While specific reasons for incomplete paperwork were not collected, it can be hypothesized that the length of time to complete “GetReady” paperwork, which included our DD screeners, as well as other standardized paperwork, may have exceeded family availability. Also, as part of the “GetReady” system, we learned that new surveys added to the system are placed at the end of the queue and the order cannot by adjusted. Thus, it is likely that the DD screeners were at the end or near the end of the package of surveys assigned to families. It may be more effective to use an institutional app for routine DD screening if it is possible to toggle the order of surveys so that the clinic can ensure that families receive the screeners earlier in their web-based paperwork.

Notably, the number of families signed up for our institutional app has increased in 3 years since becoming available (from about 25% of families enrolled to current rates). In part, the Covid-19 pandemic and related concerns [14] spurred enrollment, as telehealth functionality is built directly into the app; and many divisions at this institution, including endocrinology, have set annual goals to increase app enrollment. However, it warrants comment that to create and support a process to routinely screen for DD in youth with T1D and their caregivers using a digital health platform, it is important to select a digital health platform that families are willing to use.

### Future Directions

We plan to (1) implement a series of QI cycles to increase DD screening rates (these QI cycles will focus on current screening processes in the institutional app, as well as processes that are not app reliant if feasible, for example, integrating screening during clinic appointments); (2) expand screening to other endocrinology clinic locations within our multisite medical system; (3) create and implement a system to track follow-through on resources or recommendations sent to those with elevated DD; and (4) include options for Spanish-language speakers to receive and complete DD screening, with the eventual goal for this to be integrated into the institutional app when the app is available in Spanish. Our third goal is of particular importance given the increasing rates of T1D among Hispanic/Latinx children [1], and Hispanic youth have been identified as having the highest rates of mental health needs per Youth Risk Behavior Surveillance data [15]. The Problem Areas in Diabetes Survey—Pediatric Version (PAID-Peds) was recently normed for Spanish speakers [16], with the Spanish version of the Problem Areas in Diabetes Survey—Parent Revised (PAID-PR) also validated [17]. Improving distress screening in Spanish-speaking youth and families may assist in decreasing disparities in treatment access for mental health needs.

Relatively low rates of elevated DD were observed for the children or the caregivers in the current report. In the future, a less stringent cutoff for DD may be needed to better identify families and direct provision of referrals and resources; cut point studies may be warranted. Furthermore, given challenges previously noted specific to completion rates, it is possible that those families experiencing higher levels of distress were less likely or able to complete GetReady paperwork. Alternative methods to screen families who do not complete previsit paperwork may be necessary to improve completion rates and to identify or respond to DD. It will be important to increase buy-in at the institutional and provider level to increase opportunities to complete screening during clinical visits.

### Conclusions

DD screening is recommended by the ISPAD and the ADA as part of standards of care [2]; however, it is not consistently applied across institutions (currently, the US News & World Report review of pediatric health systems tracks only the inclusion of depression screening in youth aged 13‐18 years [18]). Given that depression is identified at lower rates than DD in populations with T1D, especially for preadolescent age groups [8,9], that DD and depression screening are not interchangeable, and that DD may play a stronger role in predicting HbA_1c_, many pediatric endocrinology clinics are missing valuable screening opportunities to direct patient care and impact health outcomes if they are screening only for depression. Our findings indicate that DD education, screening, and response can be integrated via digital platforms in a pediatric endocrinology clinic, facilitating timely treatment referral and provision of resources for those identified with distress. Of note, mean child HbA_1c_ for those with elevated DD in our sample (mean 8.04%, SD 1.72%) was higher than the mean HbA_1c_ for the larger sample of youth aged 8‐12.99 years with T1D seen in the endocrinology clinic (mean 7.75%, SD 1.46%), and higher than the clinical target of <7.0% recommended by the ADA [2]. This further emphasizes the importance of evaluating DD and providing appropriate resources and interventions in pediatric endocrinology settings.

## Abbreviations

ADA: American Diabetes Association
BPA: best practice alert
DD: diabetes distress
EHR: electronic health record
HbA_1c_: glycated hemoglobin A_1c_
ISPAD: International Society for Pediatric and Adolescent Diabetes
P-PAID-C: Parent Problem Areas in Diabetes-Child
PAID: Problem Areas in Diabetes
PAID-C: Problem Areas in Diabetes-Child
PAID-Peds: Problem Areas in Diabetes Survey—Pediatric Version
PAID-PR: Problem Areas in Diabetes Survey—Parent Revised
QI: quality improvement
T1D: type 1 diabetes
